# The different molecular forms of urine neutrophil gelatinase-associated lipocalin present in dogs with urinary diseases

**DOI:** 10.1186/s12917-014-0202-y

**Published:** 2014-08-27

**Authors:** Wei-Li Hsu, Hsiao-Chi Chiou, Kwong-chung Tung, Guillaume Belot, Anais Virilli, Min-Liang Wong, Fong-Yuan Lin, Ya-Jane Lee

**Affiliations:** 1 Graduate Institute of Microbiology and Public Health, College of Veterinary Medicine, National Chung-Hsing University, Taichung, Taiwan; 2 Department of Veterinary Medicine, College of Veterinary Medicine, National Chung-Hsing University College of Veterinary Medicine, National Chung-Hsing University, Taichung, Taiwan; 3 National Veterinary School of Toulouse, Toulouse, France; 4 Institute of Veterinary Clinical Science, School of Veterinary Medicine, College of Bio-Resources and Agriculture, National Taiwan University, No.1, Sec.4, Roosevelt Rd, Taipei, Taiwan

**Keywords:** Azotemia, Biomarker, Cystitis, NGAL

## Abstract

**Background:**

Neutrophil gelatinase-associated lipocalin (NGAL) is a useful biomarker for the early prediction of renal diseases. NGAL may exist as monomer, dimer and/or NGAL/MMP-9 complex forms in humans. In this study, the existence of various forms of NGAL in urine (uNGAL) was determined and whether these forms are related to the different urinary diseases found in dogs is further discussed.

**Results:**

Eighty-one urine samples from dogs with different forms of renal disease (41), pyuria (19) and a number of non-renal related diseases (10), as well as healthy dogs (11), were collected. uNGAL concentrations and their molecular forms in dogs were measured by ELISA and Western blot analysis, respectively. The uNGAL concentrations of dogs with pyuria (median: 15.35 ng/mL) were significantly higher than those of the healthy control animals (median: 3.92 ng/mL) (*p* < 0.01), but lower than those of dogs with renal diseases (median: 23.77 ng/mL). Each NGAL molecular form could be detected in dog urine. In particular, monomer was detected more frequently in patients with renal disease than those with non-renal diseases; while the dimer form appeared in a significantly higher percentage of cases with pyuria compared to those without pyuria. The NGAL/MMP-9 complex was found to exist not only in the patients with cystitis, but also in the cases with renal injury.

**Conclusion:**

Different molecular forms of uNGAL can indicate different origins of the urinary abnormalities. Determining the molecular forms of uNGAL present in diseased dogs may provide clinical workers with a tool that will help the early and more precise detection of different urinary diseases.

## Background

Neutrophil gelatinase-associated lipocalin (NGAL) is a glycoprotein belonging to the lipocalin family with a size of about 25 kDa [1]. NGAL is not only expressed by neutrophils but also expressed in a variety of other tissues, including non-neoplastic breast tissue, kidneys, liver, lungs, intestines, bone marrow, adipose tissue and macrophages [2]-[5]. Normally, renal NGAL is filtered via the glomerulus and then reabsorbed by the proximal tubular epithelial cells. However, when the kidneys are damaged, there is increased NGAL expression together with decreased NGAL re-absorption and these contribute to an increased NGAL concentration in both serum and urine [6]-[8].

Changes in concentrations of NGAL in both serum and urine have been proved to immediately reflect renal injury as early as 2 h after cardiac surgery [9],[10]. Moreover, these biomarkers are also considered to be good prognostic indicators for acute kidney injury (AKI) [11]-[14] and various forms of chronic kidney diseases [15],[16]. Similarly, in veterinary studies, uNGAL has been proved to be an important biomarker for dogs with different urinary diseases [10],[17]-[20].

It has been noticed that various NGAL protein forms can be detected when they are differentiated using sodium dodecyl sulfate polyacrylamide gel electrophoresis (SDS-PAGE) followed by Western blotting. NGAL monomer, with a size of approximately 25 kDa, can be observed using reducing SDS-PAGE, while NGAL dimer, with a size of approximately 45 kDa, can be detected using non-reducing SDS-PAGE [21],[22]. Furthermore, a heterodimer complex with a size of 135 kDa, where NGAL is covalently conjugated with MMP-9, can also be detected [23]. Previous studies have shown that NGAL, when secreted from neutrophils, occurs as both the 45 kDa dimer and as the 25 kDa monomer, but with the dimer being the major NGAL form. Nevertheless, under cytokine stimulation, the 25 kDa monomer form of NGAL was found to be the predominantly expressed form in the human kidney epithelial cells [24].

An increase in certain forms of uNGAL might suggest the occurrence of different diseases. For instance, increased amounts of the monomeric form of NGAL have been found in urine of human patients with AKI after cardiac surgery, while a higher proportion of NGAL dimer has been noted in human patients with urinary tract inflammation (UTI) [24]. Moreover, the NGAL/MMP- 9 complex have also been detected in a variety of tumor tissues and when humans are suffering from acute cystitis [24]-[26].

Despite the fact that the origin of the various molecular forms of uNGAL has investigated, the majority of samples analyzed as part of that series were from patients with surgery induced AKI and only five UTI cases were included in this human medical study [27]. At present, little is known about the correlation between different forms of NGAL and naturally occurring renal disease and UTI. Moreover the different molecular forms of the urinary NGAL have never been investigated in dogs. Hence, in order to obtain a better understanding of the expression profile of uNGAL and the potential clinical application of urine NGAL in dogs, the aim of the present study was to determine the presence and origin of the various different molecular forms of UNGAL together with the various factors that affect the formation of these NGAL forms, as well as concentrations of NGAL in dog urine.

## Methods

### Patients and sample collection

Samples were prospectively obtained, as part of routine diagnostics, from clinical cases admitted to the National Chung Hsing University Veterinary Teaching Hospital from September 2012 to March 2013 (IACUC approval No: 102-63). Urine samples came from catheterized and cystocentensis, and by natural voiding. The haematological parameters collected included haematocrit, white blood cell (WBC) count, neutrophil counts (segment, band) and serum/plasma biochemistry, which consisted of Blood Urine Nitrogen (BUN), creatinine, urine dipstick test (urine glucose, bilirubin, ketones, urine specific gravity, pH, urine protein, hemoglobin/blood, urine urobilinogen), White blood cells (WBC) count, and urine sediment level including red blood cells (RBCs) and WBCs in the high power field (HPF). Medical history and diagnosis were also recorded.

### Case grouping criteria

**Figure 1:**
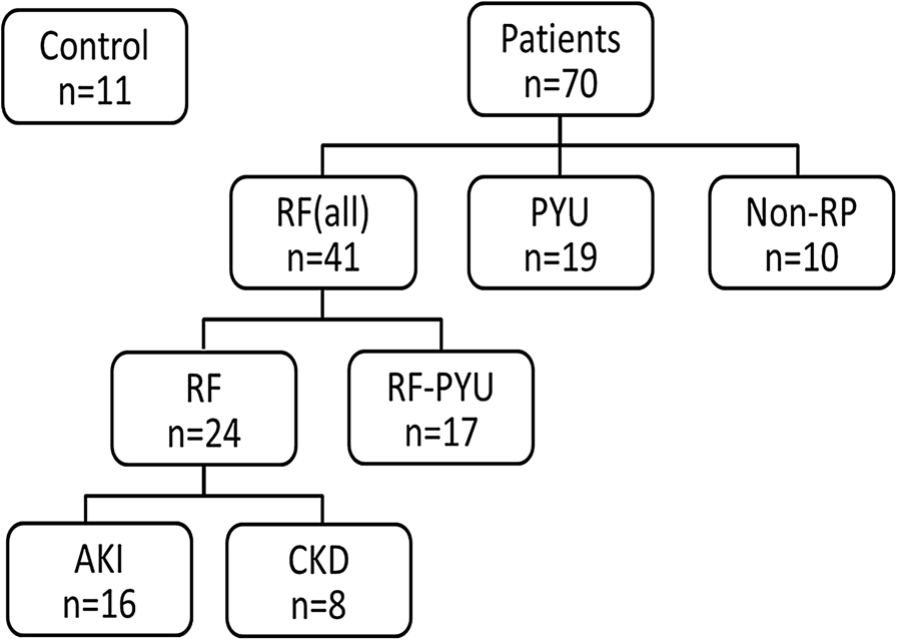
**Schematic illustration of the case groupings used in this study.** A total of 81 samples were analyzed. Based on the patients’ clinical parameters, the samples were classified into a number of different groups. These were the RF group with azotemia, the RF-PYU group with azotemia and pyuria, the PYU group with pyuria but without azotemia, the non-RP group with diseases unrelated to azotemia and pyuria; the AKI groups with acute kidney disease and the CKD group with chronic kidney disease.

The samples were divided into five groups (Figure 1). Healthy animals that were being vaccinated or undergoing health checks that had no medical history of disease, that lacked any clinical signs related to any disease, and that had BUN and creatinine levels within the normal range (BUN <8.9 mmol/L, creatinine <133 μmol/L), as well as urine leukocytes <25/μL by dipstick and WBC in urine sediment <5/HPF; these were classified as the healthy control group. The pyuria group consisted of cases without azotemia (BUN <8.9 mmol/L and creatinine <133 μmol/L) but with a WBC count >5/HPF in the urine sediment.

The dogs with clinical signs related to uremia and with a serum/plasma biochemistry showing BUN > 8.9 mmol/L, creatinine > 133 μmol/L were categorized as the renal failure (RF) group among the patients. Furthermore, on basis of the urine test results, the cases with or without pyuria criteria were categorized into the RF-PYU group, and the RF group, respectively. Finally, according to the patient’s history (less than 14 days) and diagnostic images (enlarged or small kidneys), the RF cases were divided into the AKI and chronic kidney disease (CKD) groups. The remaining ten cases that were without any urinary diseases (excluded by RF and UTI grouping) but with other diseases were classified as the non-RP group (non-RF-PYU), which included two cases of protein losing enteropathy, two cases of gastritis, two cases with hepatic disorders, one case of enteritis, one case of portal shunt, one case of bone fracture and one case of intervertebral disk disease.

### MMP 9 antibody preparation

For detection of canine MMP-9, mouse polyclonal antibody was generated. Initially, the canine MMP-9 coding region was amplified from the testicular tissue of a male dog that had been admitted to National Chung Hsing University Veterinary Teaching Hospital for neutering. Total RNA was extracted using an RNeasy® kit (Qiagen) and 2 μg of total RNA was then used as template for the generation of cDNA using a Superscript III Supermix kit (Invitrogen). The partial sequence of MMP-9 was amplified by PCR using the primer set (forward 5′-AACATATGGCCATCGCGGAGATCAGGAACTAC-3′ and reverse primer 5′-AACTCGAGGCACTGCAAAATGTCAAAG-3′); these primers amplified nucleotides 1580-2131 of the MMP-9 gene. The conditions used for the thermal cycling were an initial denaturing at 98°C for 30 seconds, and then 35 cycles of 98°C for 10 seconds (denaturing), 62°C for 30 seconds (annealing), 72°C for 30 seconds (extension); this was followed by a final extension at 72°C for 10 minutes. The resulting PCR fragment (550 base pairs) was then digested with *Nde* I and *Xho* I (the sequences of which had been introduced by primers, as indicated by the underlining) and cloned into the prokaryotic expression vector pET24a, which had been linealized with the same enzymes. A clone with an insertion of the MMP-9 sequence (plasmid MMP-9/pET24) was confirmed by restriction enzyme pattern initially and then verified by automated sequencing.

The plasmid MMP-9/pET24 was then transformed into *E. coli* (strain BL21 pLySs) and expression of the partial MMP-9 protein, which is fused with a 6-histidine tag, was induced by 0.8 mM of IPTG at 25°C overnight. Recombinant MMP-9 protein was purified using chelating Sepharose Fast Flow (GE Healthcare) and the identity of protein was confirmed by Western blot analysis using antibody against the histidine tag following the method described in a previous report [28].

Polyclonal antibodies against MMP-9 were produced using specific-pathogen-free (SPF) mice and the protocol was approved by the Institutional Animal Care and Use Committee of National University of Chung-Hsing University, permit number: 100-66. Two female BALB/c mice, purchased from National laboratory animal canter in Taiwan were kept in the same cage. Mice were initially immunized with 50 μg of the MMP-9 recombinant protein mixed with complete Freund’s adjuvant (Sigma) per mouse and this was followed by two boosters of the same dose at two-week interval. Plasma was then obtained from the immunized mice and stored at -20°C until use.

### Western blot analysis

For the reducing SDS-PAGE, pre-clarified (centrifugation at 3000 rpm for 5 min at 4°C) urine samples were mixed with 5× SDS sample buffer (0.31 M Tris, 10% SDS, 50% glycerol) and this was followed by boiling for 5 min before resolving the proteins present in the urine by 10 or 12% SDS-PAGE. For the non-reducing SDS-PAGE, the protein mix was directly loaded into gel without boiling. Subsequently, the gel was electrophoretically transferred to nitrocellulose membrane. The Western blot analysis followed the procedures described in a previous report [10]. Briefly, the filter was blocked using PBS containing 0.1% Tween-20 (PBST) and 5% skimmed milk for 1 hour at room temperature and then the filters were incubated with diluted first antibody (Ab), namely 1:800 diluted rabbit anti-canine NGAL Ab or 1:100 diluted mouse anti- canine MMP-9 polyclonal Ab. After incubation at 4°C for overnight, the membrane was washed thoroughly in PBST. This was then followed by adding 1: 10000 diluted horseradish peroxidase (HRP)-conjugated secondary antibody in PBST with 2% dried milk at room temperature for 1 hour. After washing with PBST, the membrane was developed using an enzyme-linked chemiluminescence system (GE Healthcare Bio-science Corp) and scanned on a Kodak Image Station 2000R.

Based on the existence of the distinct molecular forms of NGAL, regardless of whether there were other uNGAL forms present at the same time, all the cases were grouped into the following categories: monomer (+) and monomer (-) groups; dimer (+) and dimer (-) groups; NGAL/MMP-9 complex (+) and NGAL/MMP-9 complex (–) groups.

### Measurement of urine NGAL concentrations by ELISA

The concentration of NGAL in urine was determined by sandwich ELISA as described in our previous study [10]. Briefly, the capture antibody (1:800 diluted rabbit anti-canine NGAL antibody) was coated. After blocking, all the test samples were diluted 20-fold with PBST containing 5% dried milk. Recombinant NGAL proteins at known concentrations were used as positive controls to calibrate the system. Samples were individually added to each well and incubated at 4°C overnight. After washing with PBST, 1:3000 diluted mouse anti-canine NGAL antibody (the detector antibody) was added to the well followed by incubation at 37°C for 1 hour. Subsequently, 5,000 fold diluted HRP conjugated goat anti-mouse IgG antibody was added to the wells and incubated for 1 hour. The result was visualized using a tetramethylbenzidine (TMB) substrate kit (Clinical Science Laboratory, Inc) and the optical density (OD) was read using a microplate reader (TECAN sunrise). Each sample was tested three times independently. The OD values of the triplicates were averaged. Samples with an OD value three-fold higher than the negative control serum were considered NGAL-positive.

### Statistical analysis

SPSS 16.0 for Windows was used for data analysis. Initially, the variable dataset was assessed using the Shapiro-Wilk test. Normally distributed data are presented as mean (standard error). An analysis of variance (ANOVA), together with the Student’s t test, was applied to compare means. Non-normally distributed data are presented as median and interquartile range (IQR). The Mann-Whitney *U* test and Kruskal-Wallis Test were used for nonparametric analysis. The LSD test and Mann-Whitney U were used as *post hoc* assessments for the normally distributed and non-normally distributed data, respectively. Categorical data are presented as proportions. The *χ*2 test or the Fisher’s Exact Test was utilized to compare the datasets. Values where *p* < 0.05 are considered to be significantly different.

## Results

In this study, the uNGAL concentrations of the 81 dogs were measured and categorized in different groups (Figure 1). The cases without any urinary diseases (excluding the RF and UTI groupings), but with other diseases, were classified as the non-RP group (non-RF-PYU), which included two cases of protein losing enteropathy, two cases of gastritis, two cases with hepatic disorders, one case of enteritis, one case of portal shunt, one case of bone fracture and one case of intervertebral disk disease. Samples from the RF group (AKI and CKD) had the highest concentration of uNGAL, followed in descending order by RF-PYU, PYU, non-RP and the healthy control group. Comparing the uNGAL concentrations between groups, despite the fact that there was no significant difference between the non-RP and control group, significant differences were observed between these two groups and the other groups. For instance, the uNGAL concentrations of RF, RF-PYU, PYU, non-RP were significantly higher than those of the non-RP and the healthy control groups. Although the uNGAL level in the RF group (both AKI and CKD) was higher than control and non-RP, there was no significant difference in uNGAL level among all the groups (AKI, CKD, RF-PYU and PYU) in relation to different urinary diseases (Table 1). The AKI and RF-PYU groups showed significantly higher serum creatinine levels than CKD group, however, the serum creatinine levels of the Non-RP, control and PYU groups were lower than the CKD group (Table 1).

**Table 1:** Comparative analysis of the urinary NGAL concentrations among the different groups

**Parameter**	**Control (n = 11)**	**AKI (n = 16)**	**CKD (n = 8)**	**RF-PYU (n = 17)**	**PYU (n = 19)**	**Non-RP (n = 10)**
**uNGAL (ng/mL)**	3.92^a^ (9.05)	37.90^b^ (56.08)	18.96^b^ (11.19)	18.97^b^ (70.32)	15.35^b^ (27.663)	9.22^a^ (10.36)
**Age (year)**	6 (4)	8 (4.75)	7 (10)	5 (4)	5.5 (2.25)	4.5 (4.5)
**BUN (mmol/L)**	4.6^a^ (3.9)	49.3^d^ (53.6)	15.4^b^ (13.1)	32.5^c^ (20.5)	7.5^a^ (4.3)	6.0^a^ (4.11)
**Creatinine (μmol/L)**	97.3^a^ (44.2)	291.7^c^ (580.8)	194.5^b^ (92.8)	291.7^c^ (154.7)	106.1^a^ (44.2)	70.7^a^ (59.2)
**Urine Sp. Gr.**	1.02 (0.01)	1.019 (0.01)	1.014 (0.02)	1.015 (0.01)	1.015 (0.01)	1.015 (0)

Analysis of variance among and between the groups was performed by Kruskal-Wallis Test and Mann-Whitney T test individually and the median (IQR) is presented. Different superscript letters (a. b, c) represents a significant difference between two groups (*p* < 0.05). AKI: acute kidney injury; CKD: chronic kidney disease; RF-PYU: azotemia with pyuria; PYU: pyuria without azotemia; non-RP: diseases without any relation of azotemia and pyuria.

Urine Sp. Gr.: urine specific gravity.

**Figure 2:**
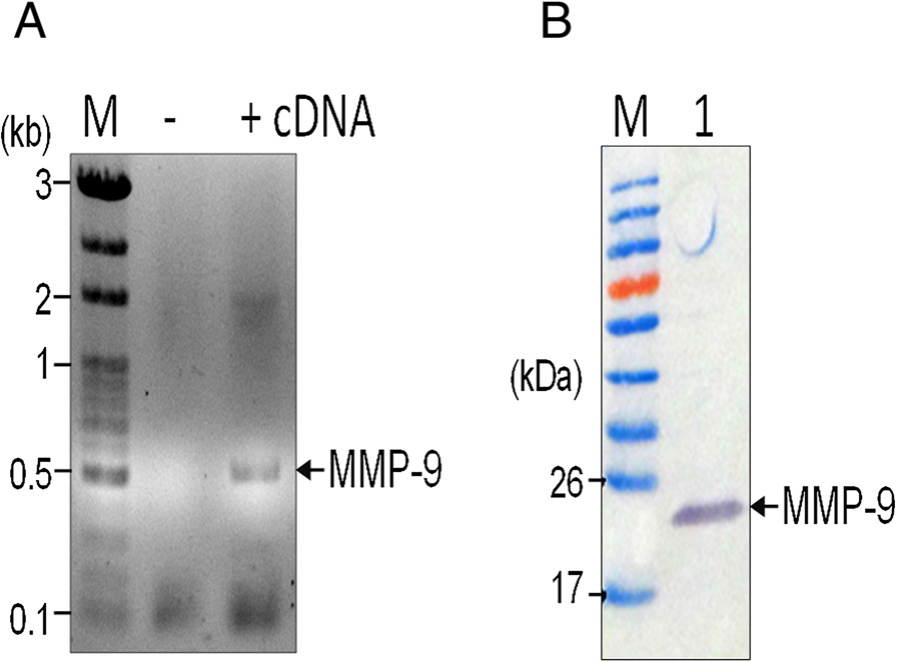
**Preparation of recombinant MMP-9 protein for mouse immunization. (A)** Partial MMP-9 coding region (nucleotide1580-2131) was amplified by RT-PCR using cDNA (lane +) generated from RNA of canine testis. **(B)** The recombinant MMP-9 with expected size of 22 kilo-Dalton (kDa) was expressed in *E. coli* and purified by metal affinity chromatography (lane 1).

**Figure 3:**
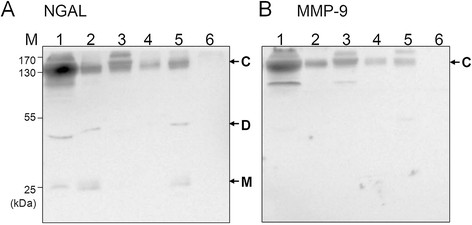
**Western blot analysis of the various NGAL molecular forms.** Urine samples representing the PYU (lane 1, 5), AKI (lane 2), non-RP (enteritis, lane 3), CKD (lane 4), and control (lane 6) groups were resolved using non-reducing SDS-PAGE followed by Western blot analysis using polyclonal antibodies against canine NGAL **(A)**, or canine MMP-9 **(B)**. Three different NGAL related proteins, as indicated by the arrowheads, were recognized by the anti-NGAL antibodies; the protein with highest molecular weight could also be detected using the anti-MMP-9 antibodies. M, D, and C indicate the monomeric NGAL, the dimeric NGAL, and the NGAL/MMP-9 complex, respectively.

Canine MMP9 antibody was successfully produced (Figure 2). As shown in Figure 3A, three different forms of uNGAL were successfully detected in all the urine samples collected from patients (Figure 3). The two forms with smaller molecular weight, namely ~25 kDa and ~50 kDa, represent the monomeric and dimeric forms of NGAL, respectively. The remaining higher molecular weight form of the NGAL related protein, with a molecular weight over 130 kDa, was then identified as the NGAL/MMP-9 complex by Western blot analysis using antibodies against canine-MMP-9 (Figure 3B).

The presence of monomeric and dimeric uNGAL was noted in 47 cases, and 20 cases, respectively (Table 2). Compared to the cases without uNGAL monomer, the dogs with monomeric uNGAL were found to have higher levels concentrations of uNGAL, BUN, creatinine, blood leukocytes including WBCs, segmented neutrophils and neutrophils (p <0.01) (Table 2); nevertheless, the increase in WBC numbers in the urine was not significant among the dogs where monomeric uNGAL was present (*p* = 0.097). Dimeric uNGAL was detected in 20 dogs. The only significantly different parameter between the dogs with dimer uNGAL and without dimer uNGAL was urine WBC numbers (*p* <0.01).

**Table 2:** Parameters with significant differences between the groups with and without monomeric uNGAL present

**Parameter**	**Monomer(+)(n = 47)**	**Monomer(-)(n = 34)**	***p*** *
**uNGAL (ng/mL)**	47.39 (23.74)	8.328 (16.16)	0.001
**BUN (mmol/L)**	21.42 (34.99)	19.76 (10.98)	0.001
**Creatinine (μmol/L)**	194.48 (194.48)	123.76 (68.95)	0.007
**WBC (/μL)**	20200 (16100)	11300 (6775)	<0.001
**Segmented neutrophil (/μL)**	17072 (13000)	8075 (6433)	<0.001
**Neutrophil (/μL)**	17460 (13500)	8316 (6540.75)	<0.001

The Mann-Whitney T test was used and the median (IQR) is presented. *p < 0.05 indicates a significant difference. Monomer (+): with monomeric NGAL; monomer (-): without monomeric NGAL.

In addition, 55/81 samples were found to contain the NGAL/MMP-9 complex (Table 3). Dogs that had the urine NGAL/MMP-9 complex present were found to have significantly higher BUN, creatinine, urinary red blood cells and urinary white blood cells, but had a lower uNGAL, compared to those dogs that lacked the NGAL/MMP-9 in the urine (*p* <0.05) (Table 3). However, no significant difference was found when the blood leukocyte counts of the two groups with and without NGAL/MMP-9 complex were compared.

**Table 3:** Parameters with significant differences between the groups with or without urine NGAL NGAL/MMP-9 complex present

**Parameter**	**Complex (+)(n = 55)**	**Complex (−)(n = 26)**	* ***p***
uNGAL (ng/mL)	16.973 (33.52)	18.697 (17.07)	0.022
BUN (mmol/L)	20.71 (34.99)	7.5 (6.6)	<0.001
Creatinine (μmol/L)	185.64 (203.32)	114.92 (53.04)	0.001
Urine RBC (/μL)	150 (225)	0 (31.25)	<0.001
Urine WBC (/μL)	25 (500)	0 (100)	0.018

The Mann-Whitney T test was used and the median (IQR) is presented. *p <0.05 indicates a significant difference. Complex (+): with NGAL/MMP9 complex; Complex (-): without NGAL/MMP9 complex.

BUN: blood urine nitrogen; RBC: Red blood cell; WBC: white blood cell; NGAL: neutrophil gelatinase-associated lipocalin; MMP9: Matrix metallopeptidase 9.

Based on the occurrence of renal failure, the whole study population (n = 81) could be divided into a RF (all) group (n = 41), and a non-renal failure group (n = 40), with the latter including the healthy Control, PYU and non-RP groups (Figure 1). Interestingly, a significantly higher frequency of monomer and NGAL/MMP-9 complex together was present in the urine of cases in the RF (all) group compared to the group without renal failure (Table 4).

**Table 4:** Comparative analysis of the proportions of cases with the three individual molecular forms of uNGAL across the different groups

	**Monomer**	**Dimer**	**NGAL/MMP-9 (+)**
**RF(all)**	73.1% (30/41)	26.8% (11/41)	50% (20/40)
**RF-**	42.5% (17/40)	22.5% (9/40)	85.3% (35/41)
***p***	0.005*	0.651	0.001*
**PYU(all)**	69.4% (25/36)	41.2% (15/36)	77.8% (28/36)
**PYU-**	48.9% (22/45)	11.1% (5/45)	60% (27/45)
***p***	0.063	0.002*	0.89
**RF**	75% (18/24)	20.8% (5/24)	79.1% (19/24)
**PYU**	68.4% (13/19)	47.4% (9/19)	63.6% (12/19)
***p***	0.633	0.065	0.245
**RF-PYU**	70.1% (12/17)	35.3% (6/17)	94.1% (16/17)
**PYU**	68.4% (13/19)	47.4% (9/19)	63.6% (12/19)
***p***	0.888	0.463	0.044*

The data was analyzed by *χ*2 test. *indicates a significant difference - found (p<.05. RF (all) azotemia; PYU(all): pyuria; RF: azotemia without pyuria; RF-PYU: azotemia with pyuria; PYU: pyuria without azotemia.

When the presence or absence of pyuria was considered, the whole study population *(*n = 81) could be divided into a PYU (all) groups (n = 36), which includes the PYU and RF-PYU groups (Figure 1), and the Pyuria (-) group (n = 45), which includes the control, RF and non-RP groups. When the presence of the various molecular forms of uNGAL were compared between the PYU (all) and Pyuria (-) groups, the result indicated that only dimeric NGAL was present at higher frequency across the PYU group (*p* = 0.002). Furthermore, there was no significant difference in terms of NGAL molecular forms between the RF and PYU groups. However, among other groups, the cases within both the RF and PYU groups had a significantly higher proportion of cases with the uNGAL/MMP-9 complex present (Table 4).

## Discussion

In this study, anti-canine NGAL and anti-canine MMP-9 polyclonal antibodies were produced in our laboratory and these were successfully used to detect the various different molecular forms of urine NGAL in dogs by Western blotting using non-reducing conditions. Similar to human patients [21],[23], uNGAL in dogs has three forms, namely a 25 kDa monomer, a 50 kDa dimer and a 130 kDa NGAL/MMP-9 heterodimer complex. Overall, the presence of monomer uNGAL was found to be significantly associated with abnormal levels of serum BUN and creatinine. Furthermore, the proportion of cases with uNGAL monomer was also significantly different between RF negative and the RF all groups; thus the increase of uNGAL monomer appears to be related to kidney injury, which indirectly indicates that canine uNGAL monomer is likely to have originated from the kidneys. However, the cases with monomeric uNGAL were found to be associated with an increased presence of urine blood leukocytes in this study. In humans, blood neutrophils have been reported to show a positive correlation with NGAL concentration in serum but not to be related to urine NGAL levels [29]. In this study, the cases with higher blood leukocytes also had RF, and therefore the presence of renal diseases may have influenced the results. To investigate this, a comparative analysis of uNGAL concentration and blood WBC, as well as neutrophils, across the non-RP and control groups was carried out and this indicated that both WBC and neutrophils were not significantly related to uNGAL (data not shown). This implies that the concentration of uNGAL is affected significantly by urinary tract diseases.

An increased uNGAL level has been reported to be an early diagnosis indicator of urinary tract infection [30]; additionally, white blood cells in urine have been found to be correlated with an increase in uNGAL concentration [31]. Consistent with the above, in the present study, the uNGAL concentration in dogs with pyuria (median: 15.35 ng/ml) was found to be significantly higher than that in the control group (3.92 ng/ml), which indicates that pyuria indeed increases the level of uNGAL. Moreover, among the three forms, only the dimeric uNGAL was associated with urine WBC; the dimeric form of NGAL appears to be the major form of uNGAL present in pyuria patients. An increase in the dimeric form of uNGAL may be a useful way of differentiating disease from kidney origin, where there is secretion of monomeric NGAL.

The appearance of the uNGAL/MMP-9 complex was found to be related in the present study to the presence of increased creatinine, proteinuira, and increased numbers of urine leukocytes. The uNGAL/MMP-9 complex has been reported to be related to the presence of cystitis in children [31], but in this study, the uNGAL/MMP-9 complex appeared not only to be present in samples from the PYU groups but also in samples from the RF without PYU group. Furthermore, the RF-PYU groups had a higher proportion of uNGAL/MMP9-complex than the PYU alone group. Inflammation has been shown to stimulate the expression of MMP-9 [32], and, in the present study, the cases with renal failure also had higher leukocytes counts, and therefore the increase in the presence of the uNGAL MMP9 complex in the renal failure group seems rational. Based on the above findings, the role that the uNGAL/MMP9 complex plays in non-renal diseases needs to be investigated further.

In this study, a no-RP group was included to evaluate the possible contribution of other diseases to the uNGAL increase and this preliminary analysis showed that uNGAL does not seem to be affected by other diseases. However, the RF group was based on azotemia; as a result, prerenal factors that contribute to any tubular injury and were responsible for the elevation of uNGAL, cannot be excluded.

Although information on how UTI affect uNGAL concentrations is limited, it has been shown that, among humans, the cut-off point for uNGAL in AKI patients (50 to213 ng/mL) is higher than that in UTI patients (20 ng/mL) [30]. However, in dogs, despite higher uNGAL levels being found in AKI cases, the increase of uNGAL is not significant between the AKI cases and the cases with pyuria. The possible reasons may be as follows. First, our definition of AKI is based on the level of serum creatinine, which is known to increase later than uNGAL [33], this might have led to missing the peak uNGAL level. Second, AKI can also be defined as a rise in creatinine level from baseline; however without knowing the baseline of serum creatinine level for each case, even though the serum creatinine concentration might be within normal range, the possibility of renal injury being responsible for any rise in uNGAL among PYU cases cannot be ruled out.

## Conclusions

Our results indicate that urinary NGAL concentration alone does not seem to be able to differentiate the various different types of urinary diseases, nevertheless, both ELISA and Western blot approaches can be used to explore the changes in uNGAL and a combination of these approaches seems to be a promising way of evaluating urinary diseases. Thus, while an increase in uNGAL can imply urinary abnormality, a simultaneous differential analysis of the molecular forms of NGAL present in urine is needed in order to understand comprehensively the origin of the various forms of uNGAL.

## Abbreviations

AKI: Acute kidney disease

ANOVA: Analysis of variance

CKD: Chronic kidney disease

HPF: High power field

IQR: Interquartile ranges

MMP-9: Matrix metallopeptidase 9

NGAL: Neutrophil gelatinase-associated lipocalin

PBST: Phosphate buffered saline containing 0.1% Tween-20

RBC: Red blood cell

SDS-PAGE: Sodium dodecyl sulfate polyacrylamide gel electrophoresis

UTI: Urinary tract inflammation

WBC: White blood cell count

## Competing interests

None of the authors of this paper has a financial or personal relationship with other people or organizations that could inappropriately influence or bias the content of the paper. The authors declare that they have no competing interests.

## Authors’ contributions

YL and WH designed the experiments, analyzed the data and drafted the manuscript together. HC, FL, GB and AV performed the experiments. KT and MW helped to draft the manuscript. All authors read and approved the final manuscript.
